# Correlation between alveolar cleft morphology and the outcome of secondary alveolar bone grafting for unilateral cleft lip and palate

**DOI:** 10.1186/s12903-022-02265-4

**Published:** 2022-06-22

**Authors:** Xinlei Yu, Yiping Huang, Weiran Li

**Affiliations:** Department of Orthodontics, Peking University School of Stomatology, Zhongguancun South Road 22, Haidian District, Beijing, 100081 People’s Republic of China

**Keywords:** Alveolar bone grafting, Cleft lip, Cleft palate, Orthodontics, Cone-beam computed tomography

## Abstract

**Background:**

Secondary alveolar bone grafting (SABG) is an integral part of the treatment for cleft lip and alveolus and cleft lip and palate. However, the outcome of SABG was not satisfactory as expected, factors that affecting the outcome were still controversial. The aims of this study were to summarize a new method for the classification of alveolar cleft morphology in patients with unilateral cleft lip and alveolus or unilateral cleft lip and palate, to evaluate the correlation between the morphology and SABG outcomes, to identify factors that might predict the outcomes.

**Methods:**

The characteristics of the cleft morphologies of 120 patients who underwent SABG were observed using the preoperative Cone-Beam Computed Tomography (CBCT) images. 52 patients who had CBCT scans performed at least 6 months after SABG were included for the evaluation of outcomes. Both categorical and continuous evaluation methods were measured. Correlations between cleft morphology and SABG outcomes were assessed using the Pearson correlation coefficient in SPSS 27.0.0.

**Results:**

A new method for the classification of cleft morphology was summarized:type I, prism type (labial defect size ≥ palatal defect size; nasal defect size ≥ occlusal defect size); type II, prism’ type (labial defect ≥ palatal defect; nasal defect < occlusal defect); type III, inverted prism type (palatal defect ≥ labial defect); type IV, funnel type (presented as a significantly narrow defect area in the middle towards the vertical dimension); and type V, undefinable (extremely irregular morphology can’t be defined as any of the above types). Categorical evaluation showed 8 failure, 11 poor, 12 moderate, and 21 good results, while the average bone filling rate was 59.24 ± 30.68%. There was a significant correlation between the cleft morphology and categorical/continuous evaluation outcome (*p* < 0.05).

**Conclusion:**

The new method for the classification of alveolar cleft morphology summarized in this study was comprehensive and convenient for clinical application. Both categorical and continuous methods should be used for radiographic assessments in patients undergoing SABG. The chances of a successful procedure might be better when the patient has type I or IV morphology, in which the shape is like a funnel in the relatively palatal or occlusal area towards the vertical dimension. A relatively great amount of bone resorption was observed in most patients.

*Trial registration* Chinese clinical trial registry; registration number: ChiCTR2100054438.

## Background

Secondary alveolar bone grafting (SABG) is an integral part of the treatment for cleft lip and alveolus (CLA) and cleft lip and palate (CLP) [1]. SABG stabilizes and restores the continuity of the alveolar bone; provides bony matrix for eruption of the teeth adjacent to the cleft; supports orthodontic tooth movement; improves the outcome of nose repair by supporting the alar base; and closes the oronasal fistula to improve pronunciation [1–5]. Orthodontic movement requires adequate position and volume of bone bridge in the cleft area [6]. It is essential to evaluate patients for the size of the alveolar cleft defect, bone level at the adjacent teeth before SABG, and adequacy of bony fill of the defect, stability of bone for orthodontic tooth movement after SABG [7]. However, factors that affecting the outcome of SABG were still controversial, especially the individual cleft defect differences before surgery.

Studies of the correlation between the initial cleft defect and the outcomes of SABG showed varied results, but a moderate or strong correlation was rarely reported [8]. Most studies reported no significant correlation between the initial cleft size and the bone fill rate (BFR) [7, 9, 10]; Similar discrepancies have also been reported for the correlations between SABG outcomes and parameters such as cleft width, cleft type, and the characteristics of cleft-side lateral incisors [2, 11, 12].

The morphology of the alveolar bony defect is complex and irregular. Computed Tomography (CT) images can identify the alveolar cleft site by 3D reconstruction of pre- and post-SABG alveolar clefts. Some scholars have suggested that an individualized approach based on cleft morphology should be used to identify the site of the cleft defect instead of strict adherence to a pre-established protocol [3]. Many studies have paid attention to the morphology of the cleft defect in recent years, some studies evaluated the postoperative morphology of the cleft using two-dimensional (2D) linear measurements to visualize the location of bone resorption [13]. Garib et al. assessed the mesial and distal alveolar bones of the maxillary canines after SABG to describe the morphology of bone support [14]. Brudnicki et al. analysed the location and morphology of bone resorption after SABG [13]. However, there are several limitations to former studies: morphology was described based on cephalometric variables and 2D evaluation and although 3D assessment has already been conducted [15], there was no relevant research focused on the preoperative morphology of the cleft defect, no certain morphological description had been summarized, and whether there was a correlation between the cleft morphology and SABG outcomes remain unknown.

Three-dimensional (3D) imaging is useful for the diagnosis of alveolar cleft defect and assessment of SABG outcomes. 3D radiographic assessments can be divided into continuous evaluation methods and categorical evaluation methods. The most commonly used indices of continuous evaluation methods are the rates of bone filling and resorption [16], and the calculation of graft volume using CT has been confirmed to be reliable [17–19]. However, there is no consensus on the gold standard for defining how much fill rate is a success, and whether the site of bone loss will cause difficulty in orthodontic tooth movement cannot be identified, which reduces the clinical significance [20]. In recent years, researchers have presented categorical evaluation methods based on 3D images that describe the site of grafted bone [2, 21–24]. Most previous reports used the alveolar height and thickness after SABG to determine the categorical outcomes [14, 20, 24–26], but there was limited scope of application depend on canine eruption status, and failed to present the generalization of the measurements to evaluate the final result of SABG as well. Stasiak et al. [27] presented a novel method which solved the above limitations and considered the potential of root resorption. Certain position of bone resorption can be observed via this method and give guidance for further orthodontic movement. Therefore, a combination of continuous and categorical evaluation methods that incorporates successful orthodontic movement might be beneficial to a more comprehensive evaluation of SABG outcomes.

The aims of this study are (1) to summarize a new method for the classification of alveolar cleft morphology; (2) to evaluate the correlation between the cleft morphology and SABG outcomes; (3) to identify factors that might predict SABG outcomes.

## Methods

### A new method for the classification of alveolar cleft morphology

120 consecutive patients with unilateral CLA (UCLA) or unilateral CLP (UCLP) who underwent SABG at the Department of Oral and Maxillofacial Surgery, Peking University School and Hospital of Stomatology between 2017 and 2019 were included. All patients underwent SABG with autogenous cancellous bone harvested from the iliac crest, and one experienced surgeon was responsible for all operations. The surgical technique of SABG described by Boyne and Sands was used in these patients [28], the cleft was exposed through both vestibular and palatal approaches, and cancellous bone was grafted to the entire cleft space. Approval was obtained from the ethics committee of the Peking University School and Hospital of Stomatology (reference number: PKUSSIRB-202163059), and informed consent was obtained from all patients.

### Inclusion criteria

1. Age ≥ 8 years
2. Non-syndromic unilateral alveolar cleft with or without cleft palate
3. Lip repair be performed in the first 6 months after birth, and palate repair be performed during the first year of the infant’s life
4. Cone-beam computed tomography (CBCT) performed before SABG
5. Autogenous cancellous iliac bone used for the grafted bone

### Exclusion criteria

1. Previous alveolar bone graft
2. Application of orthodontic treatment or gingivoperiosteoplasty before SABG
3. Any severe systemic disease such as rickets or osteoporosis

Characteristics of the cleft morphologies were observed using the preoperative CBCT images from these patients, in order to summarize a new classification method which can characterize the cleft morphology and classify all 3D findings.

### Evaluation of the SABG outcome

Based on the included patients above, we further screened and selected 52 patients who had CBCT scans performed at least 6 months after SABG. Approval and informed consent were obtained (PKUSSIRB-202163059).

### Radiographic data

All patients underwent CBCT (Imaging Sciences International/17-19DX) within the 3 days prior to surgery, 52 patients underwent again at least 6 months after surgery. The imaging conditions were 120 kV, 5 mA, pixel size of 0.3 mm, and field of view (FOV) of 160.8 mm*160.8 mm. CBCT images were reconstructed with voxel dimension, and imported to the 3D analysis software (Mimics 21.0, Materialise, Leuven, Belgium) for volumetric and other analyses. The average age of the included patients at the time of SABG was 12 years (range 8–18 years).

#### Categorical evaluation method

A method presented by Stasiak et al. [27] to qualitatively evaluate the SABG results was applied. Standardization was obtained after reorientation of the images according to the long axes of central incisors on the corresponding side. The cementoenamel junctions were points of reference to establish the position of four assessment levels: 3 mm, 5 mm, 7 mm, and 9 mm. The cementoenamel junction point was set at the most apical point of the enamel on the incisor’s midsagittal cross-section. Firstly, an assessment of the presence or lack of the bone bridge due to the continuous investigation of the areas was conducted. Secondly, a classification of the bone was performed at the adequate levels in the narrowest points of the alveolar bone between canines and central incisors. Score 0: no alveolar bone bridge; score 1: thickness of the alveolar bone bridge < 1/2 of the labiolingual width of the central incisor’s root; score 2: thickness of the alveolar bone bridge ≥ 1/2 of the labiolingual width of central incisor’s root and less than the labiolingual width of central incisor’s root; score 3: thickness of the alveolar bone bridge amounts to at least the labiolingual width of central incisor’s root. The final step involved summing all the scores on each side to obtain a general assessment of the bone architecture according to the interval scale: 0, failure; 1–4, poor results; 5–8, moderate results; and 9–12, good results. Moreover, in cases of severe central incisor root resorption, an assessment according to the horizontal scale was performed at the adequate level (9 mm) but with comparison with the root diameter measured 0.5 mm beneath the apex.

#### Continuous evaluation method: bone fill rate (BFR)

**Fig. 1:**
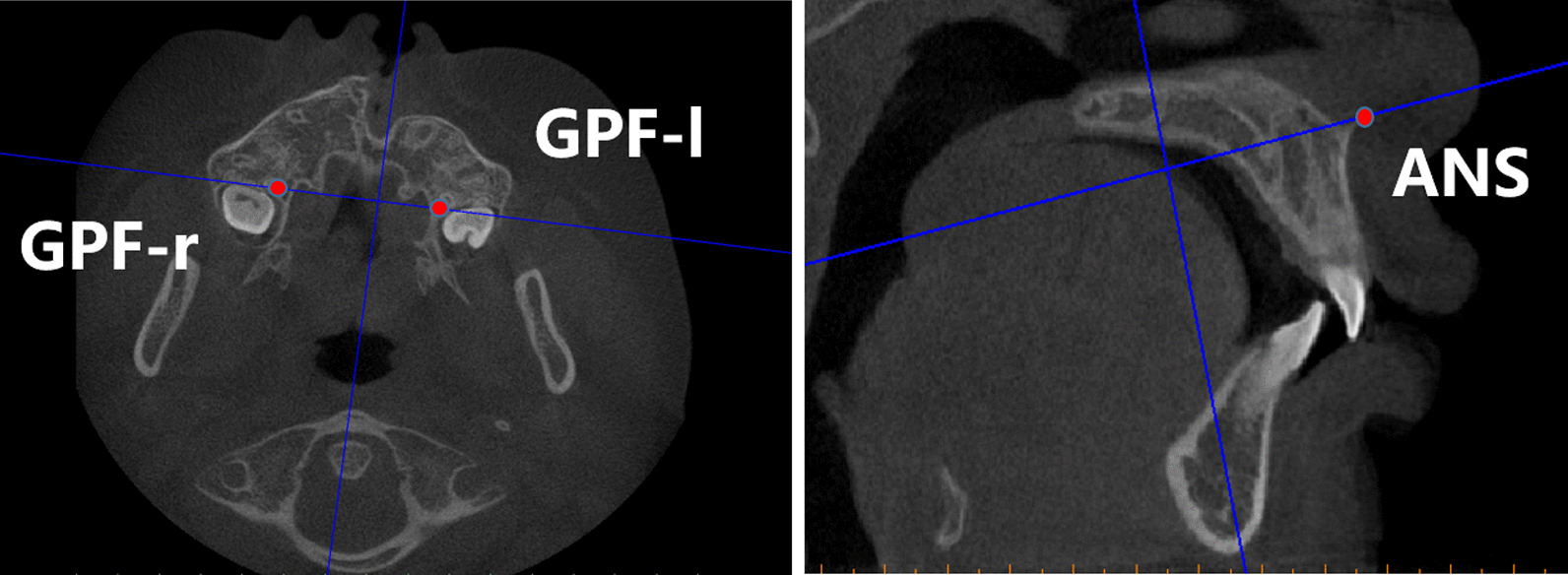
(Left) Greater palatine foramen point on the left side (GPF-L), greater palatine foramen point on the right side (GPF-R) and (Right) anterior nasal spinal point on the cone beam computed tomography axial and sagittal planes

**Fig. 2:**
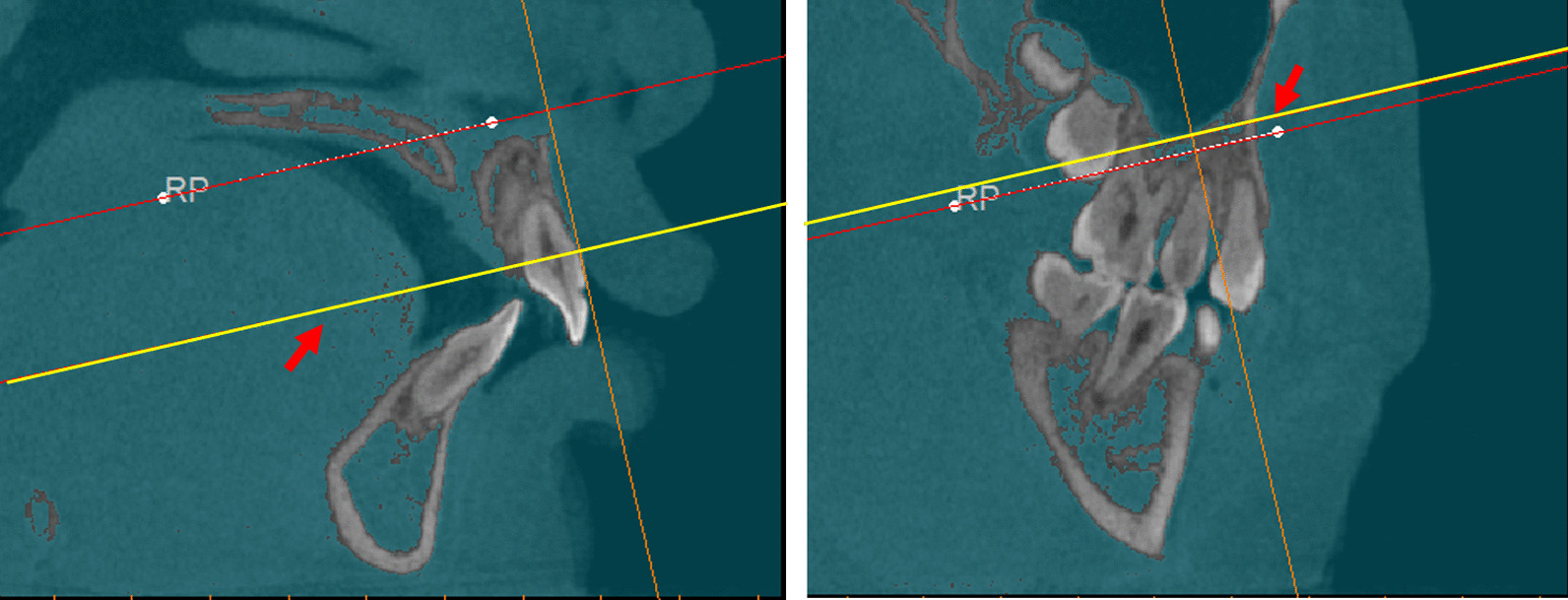
The reference plane was defined as a plane passing through the anterior nasal spinal point, greater palatine foramen point on the left side (GPF-L), and greater palatine foramen point on the right side (GPF-R). (Left, arrow) The inferior and (Right, arrow) superior planes were horizontal and parallel to the reference plane, defined to identify the upper and lower margins of the alveolar defect based on the reference plane. The inferior plane passed through the labial cemento-enamel junction of the central incisor on the cleft side, and the superior plane passed through the most inferior margin of the contralateral nostril floor in the coronal plane where canines were seen

**Fig. 3:**
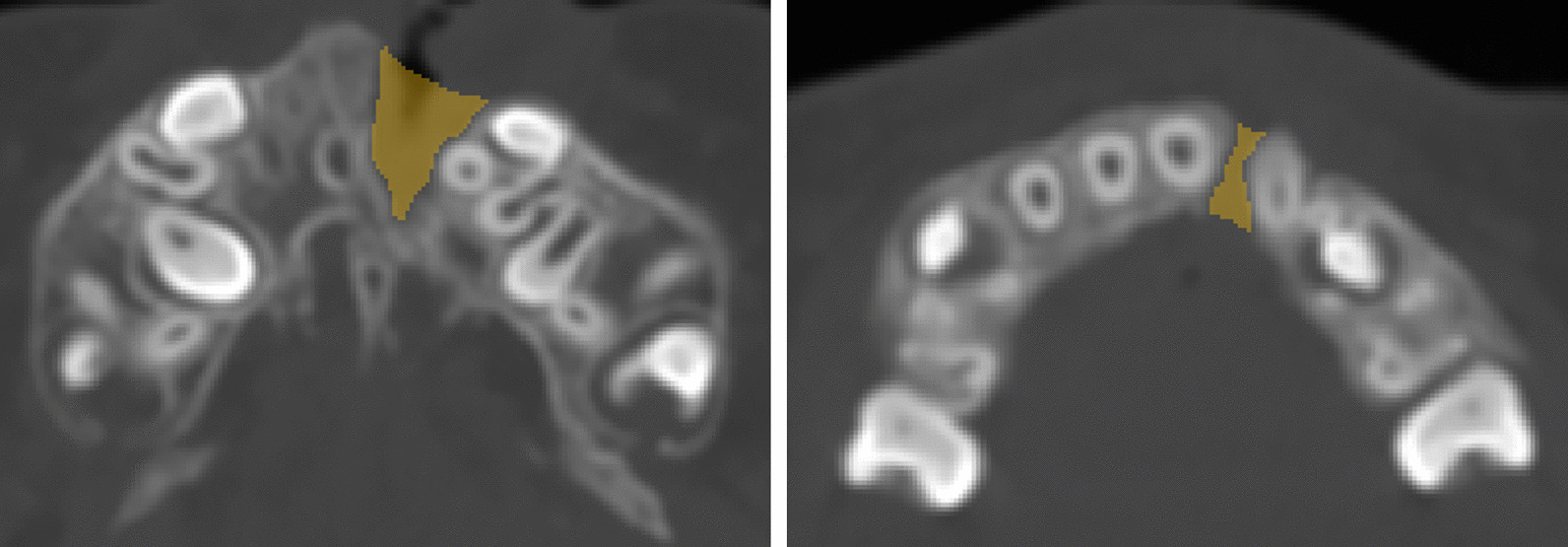
The labial and palatal margins were outlined and erased manually using the Edit mask tool, and the final mask were segmented after the Region grow algorithm. (Left) nasal side and (Right) occlusal side

**Fig. 4:**
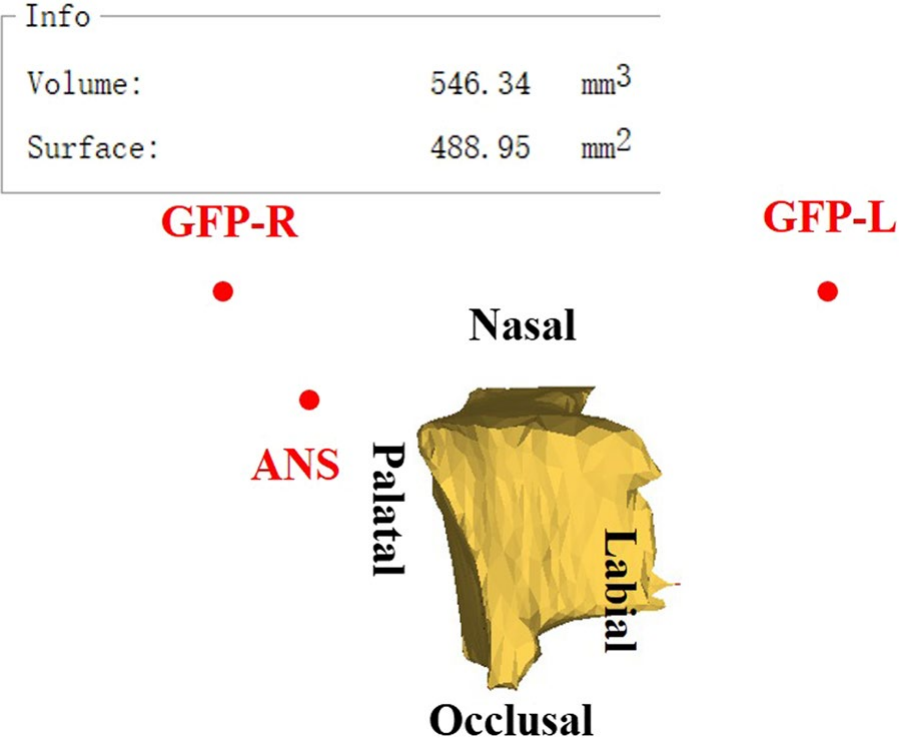
The medial view of a cleft model with landmarks after 3D reconstruction and its preoperative cleft volume (VOLpre)

A combination method presented by Feng et al. and Linderup et al. [29, 30] to calculate the volume of the cleft defect was used. Bilateral greater palatine foramen points and anterior nasal spinal point were used as landmarks for the reference plane (Fig. 1). The reference plane was made horizontal, and the images were reoriented along this reference plane to standardize among patients. The inferior and superior planes were horizontal and parallel to the reference plane, defined to identify the upper and lower margins of the alveolar defect. The inferior plane passed through the labial cemento-enamel junction of the central incisor on the cleft side, and the superior plane passed through the most inferior margin of the contralateral nostril floor in the coronal plane where canines were seen (Fig. 2). Then, an appropriate threshold of grey value (approximately 300–3800 HU) was determined to distinguish the cleft area from bilateral cortical bone based on the profile line defined by the operator, the labial and palatal margins were manually outlined and erased using the Edit mask tool between the inferior and superior planes, and the final mask were segmented after the Region grow algorithm (Fig. 3). The images were checked twice to prevent inaccurate distinction in each slice, so as to ensure the manually outlined and erased model line will not influence quantitative results afterwards. Finally, a 3D cleft model was reconstructed and separated, the preoperative cleft volume (VOLpre) was calculated automatically (Fig. 4). The same method was used to calculate the postoperative cleft volume (VOLpost) at the 6-month follow-up. The bone fill rate was calculated using the formula (VOLpre – VOLpost) / VOLpost.

### Parameters of the cleft defect

CBCT scans obtained before SABG were assessed for the morphology, volume, and type of cleft. CBCT scans obtained after SABG were evaluated for the residual defect volume and SABG outcomes. The samples were measured twice within a 3-month interval by two orthodontists separately who were trained to manage patients with CLA/CLP.

### Reliability of the recordings

Blind method was adopted in the research process as well as the subgroup analysis. All measurements were obtained by two trained researchers independently. The reliability and inter-rater reproducibility of the cleft defect measurements were determined from randomized duplicate recordings in 52 UCLA/UCLP patients. Intraclass correlation and kappa coefficients were used to determine the inter-rater agreement.

### Statistical analysis

Statistical analyses were performed using SPSS 27.0.0 (IBM Corp, Armonk, NY, USA). All measurements were obtained by two trained researchers independently. The mean and range were calculated for all variables. Spearman’s rank-order correlation coefficient was calculated for correlations among the categorical classification scale for the cleft morphology, categorical evaluation method, and BFR. The correlations among the VOLpre, patient age, and BFR were assessed using the Pearson correlation coefficient.

## Results

### A new method for the classification of alveolar cleft morphology

**Fig. 5:**
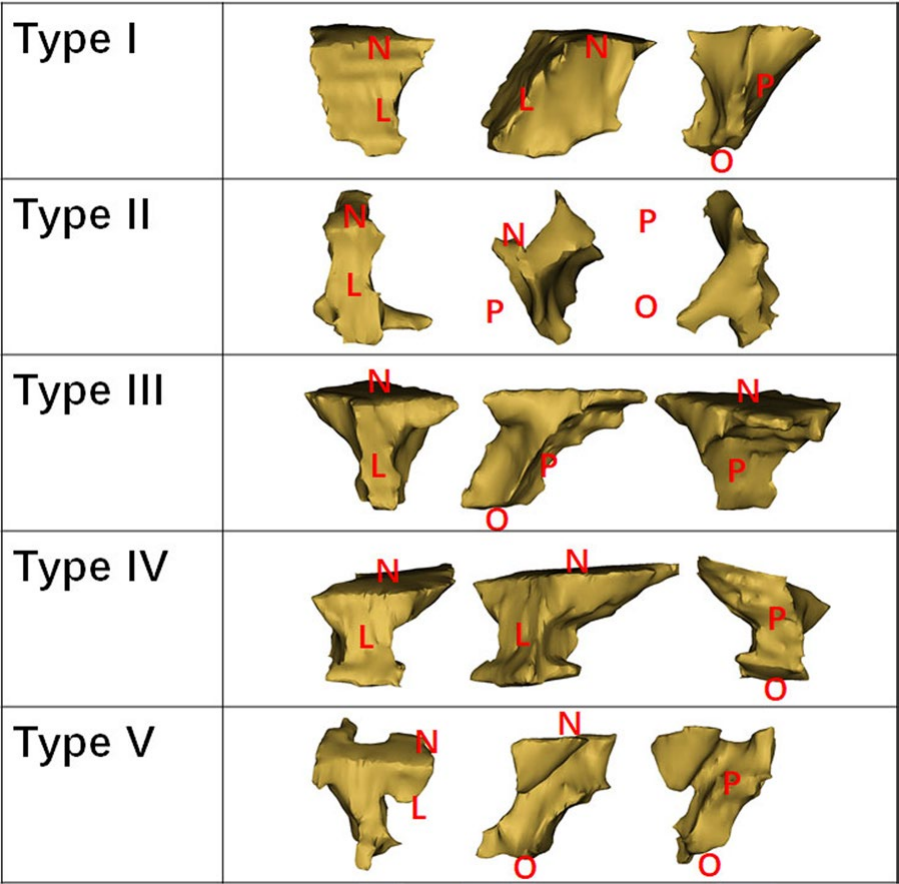
A new method for the classification of alveolar cleft morphology consisting of five types: type I, prism type (labial defect size ≥ palatal defect size; nasal defect size ≥ occlusal defect size); type II, prism type (labial defect ≥ palatal defect; nasal defect < occlusal defect); type III, inverted prism type (palatal defect ≥ labial defect); type IV, funnel type (significantly narrow defect in the middle towards the vertical dimension); and type V, undefinable (extremely irregular morphology)

After reorienting the images along the reference plane described above (Fig. 1), the morphologies of the initial alveolar cleft defect were reviewed based on 3D reconstruction in 120 preoperative CBCT images. The defect sizes were compared along the labial-palatal axis and the nasal-occlusal axis according to reference plane. The majority of the scans exhibited a larger labial defect size compared with the palatal size and larger nasal defect size compared with the size of the occlusal part. Relatively few scans showed the opposite findings. However, the shape was not regular for all clefts. Many clefts had a relatively irregular shape and narrowing in the middle part of the cleft defect, causing the cleft morphology to appear like a funnel. Because these clefts accounted for a significant proportion of all clefts, they were divided into a separate group. Based on these measurements, a new method for the classification of alveolar cleft morphology was summarized: type I, prism type (labial defect size ≥ palatal defect size; nasal defect size ≥ occlusal defect size, presented as a widest cleft defect located at the most labial, nasal area, and gradually narrowed towards both the vertical and horizontal dimension, while the narrowest part located at the most palatal, occlusal area); type II, prism’ type (labial defect ≥ palatal defect; nasal defect < occlusal defect, presented as a widest cleft defect located at the most labial, occlusal area, and gradually narrowed towards the vertical and horizontal dimension, while the narrowest part located at the most palatal, nasal area); type III, inverted prism type (palatal defect ≥ labial defect, presented as a wider cleft defect at the palatal side compared with the labial side and cleft width gradually increased along the labial-palatal axis, without a significantly narrow defect area in the middle towards the vertical dimension); type IV, funnel type (presented as a significantly narrow defect area in the middle towards the vertical dimension); and type V, undefinable (extremely irregular morphology can’t be defined as any of the above types; Fig. 5, Table 1).

**Table 1:** Classification of the alveolar cleft morphology in 120 patients with cleft lip and alveolous or cleft lip and palate

Type	
I	40
II	4
III	16
IV	48
V	12
Total clefts	120

### Evaluation of the SABG outcome

#### Descriptive measures

The characteristics of the 52 patients were summarized in Table 2. The age of the subjects at the time of SABG ranged from 8 to 18 years, of which 23 were 9–11 years. The study group consisted of 13 female (25%) and 39 male (75%) patients. There were 26 right (50%) and 26 left (50%) clefts. The average VOLpre was 0.82 ± 0.37 mL, ranging from 0.16 to 1.92 mL. The average VOLpost was 0.36 ± 0.33 mL, ranging from 0.013 to 1.64 mL.

**Table 2:** Subject Information

Total cases (Alveolar clefts)	52
Age at grafts	12.31 ± 3.08y
Sex (M:F)	39:13
Cleft type	
Left:Right	26:26
UCLA:UCLP	11:41

### Interobserver reproducibility

There were excellent interobserver reproducibility (intraclass correlation coefficient = 0.905; *p* < 0.001) and good agreement in the categorical evaluation (kappa = 0.763) of the SABG outcomes.

#### Categorical evaluations

High individual variability was found. The final results showed 8 failure (15.4%), 11 poor (21.1%), 12 moderate (23.1%), and 21 good (40.4%) results of SABG procedure (Table 3). The alveolar bone was classified as good in all patients on the noncleft side.

**Table 3:** Total score of cleft and noncleft side patients

Side	Number of patients												
	Failure	Poor				Moderate				Good			
	0	1	2	3	4	5	6	7	8	9	10	11	12
Cleft	8	2	3	4	2	4	3	2	3	2	4	6	9
Noncleft	0	0	0	0	0	0	0	0	0	0	0	0	52

On the cleft side, 69 sites were classified as 0, 30 sites as 1, 21 sites as 2, and 88 sites as 3. Statistics showed that 60.9% score = 0 sites occurred at 3 mm or 5 mm measurement heights; whereas 64.8% score = 3 sites occurred at 7 mm or 9 mm measurement heights. On the noncleft (control) side, all sites were classified as 3 (Table 4). The measurement modification at the level of 9 mm was used in 5 patients due to the cleft side central incisor’s root resorption.

**Table 4:** Intergroup comparisons at different measurement levels

Measurement heights	Number of patients							
	Cleft scores				Noncleft scores			
	0	1	2	3	0	1	2	3
3 mm	21	8	12	11	0	0	0	52
5 mm	21	5	6	20	0	0	0	52
7 mm	13	11	3	25	0	0	0	52
9 mm	14	6	0	32	0	0	0	52

#### BFR

The average BFR was 59.24 ± 30.68%, ranging from 0.39 to 99.16%. The median number was 67.74%. Residual bone ratio was less than 50% after 6-months follow-up in 19 patients (36.5%).

#### Correlation between initial cleft defect morphology and SABG outcome

**Fig. 6:**
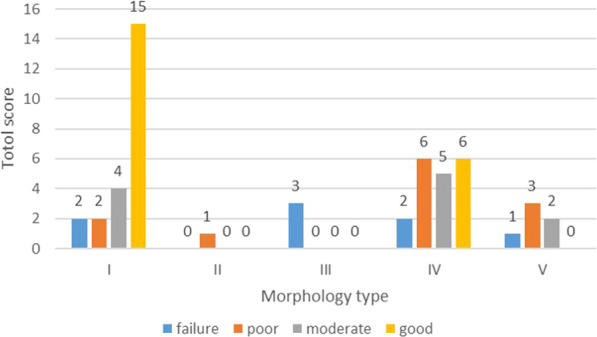
Plot with the total scores of all 52 patients according to the new method for the classification of alveolar cleft morphology. Type I, prism type (labial defect size ≥ palatal defect size; nasal defect size ≥ occlusal defect size); type II, prism’ type (labial defect ≥ palatal defect; nasal defect < occlusal defect); type III, inverted prism type (palatal defect ≥ labial defect); type IV, funnel type (presented as a significantly narrow defect area in the middle towards the vertical dimension); and type V, undefinable (extremely irregular morphology can’t be defined as any of the above types)

There was a significant correlation between the initial cleft defect morphology and categorical/continuous evaluation outcome (*p* < 0.05; Table 5, Fig. 6). Using the new classification method described above, there were 23, 1, 3, 19, and 6 patients with type I, II, III, IV, and V morphology, respectively. In 23 patients whose cleft morphology been classified as type I, the outcome of SABG showed 15 good, 4 moderate, 2 poor and 2 failure results via categorical evaluation method. While type II morphology was relatively rare compared with other types, with only 1 patient whose outcome was poor. Type III exhibited the worst results with all 3 patients been classified as failure, which reflects non-existence of bone bridge at any horizontal levels. Type IV was a rather common type, but the results varied within the type, with 6 good, 5 moderate, 6 poor and 2 failure results. And for the 5 patients with extremely irregular cleft morphology only can be classified separately as type V, the outcome included 1 failure, 3 poor and 2 moderate results. Furthermore, statistics showed that all good results came from type I or type IV cleft morphology, while 81.8% moderate results came from type I or type IV.

**Table 5:** Morphology, type, side of the cleft and age,sex of patient for SABG outcomes evaluated using Spearman's/Pearson's correlation analysis

		Morphology	Morphology(subgroup)	VOLpre	Type	Side	Sex	Age
Continuous evlaution method (BFR(%))	Correlation coefficient	− 0.353*	− 0.505**	0.002	− 0.041	0.056	0.090	− 0.123
	p value	0.010	0.001	0.988	0.774	0.691	0.525	0.387
Categorical evaluation method	Correlation coefficient	− 0.373**	− 0.472**	0.016	0.055	0.051	0.022	− 0.261
	p value	0.007	0.001	0.908	0.697	0.719	0.878	0.062

VOLpre, preoperative cleft volume; *Correlation is significant at the 0.05 level;**Correlation is significant at the 0.01 level

### Subgroup analysis

- Subgroup analysis was performed on 23 patients aged 9–11 years. Statistical analysis results also shown that there was a significant correlation between the initial cleft defect morphology and categorical/continuous evaluation outcome (*p* < 0.05).
- The outcome of SABG in patients with type I and type IV morphology, in which the shape is like a funnel in the relatively palatal or occlusal area towards the vertical dimension were better than patients with type II, III, V and type IV morphology, in which the shape is like a funnel in the relatively labial or nasal area towards the vertical dimension (*p* < 0.01; Table 5).

#### Correlations of the initial cleft size and dental parameters with SABG outcome

The outcomes of SABG did not have any significant correlations with the alveolar cleft parameters, including the initial bone defect volume, patient age or sex, cleft type or side (Table5).

## Discussion

The new method for the classification of alveolar cleft morphology presented in this study was able to cover all kinds of morphology types in clinical, and the intuitive classification pattern could be convenient for clinical application. In the past, periapical radiographs were the most frequently used 2D imaging modality to assess cleft morphology, and many radiographic scales have been developed for cleft defect description [4, 16, 21, 23, 29]. Cleft width was the most commonly used parameter of initial cleft defect in literature, though some studies assessed cleft width in different levels, but it could only reflect the characteristics of cleft defect in vertical dimension, which neglect the labial-palatal direction condition. While the importance of the cleft defect status in labial-palatal direction pre- and post-operatively has been emphasized because a high amount of resorption was normally present in the horizontal plane [31]. Therefore, the new method for the classification of alveolar cleft morphology in this study was summarized after observing a large sample of preoperative CT images of UCLA/UCLP patients, and considering the limitations of the previous researches simultaneously. Based on the initial cleft defect reconstructed from 3D images, the morphology was divided into five types according to the different characteristics in labial–palatal and nasal–occlusal dimensions.

Significant correlation between cleft morphology and SABG outcomes suggested that the chances of a successful procedure might be better when the patient has type I or IV morphology, in which the shape is like a funnel in the relatively palatal or occlusal area towards the vertical dimension. Three cases in our study showed a relatively high BFR (62.15%, 67.38%, and 80.54%), but CBCT showed that the bone bridge existed only in the apical 1/4 area and the nasal area. Although the width of the defect was reduced after SABG, there was no effective bone bridge at other levels, which explains the failure of a successful outcome in patients with a relatively high BFR. The morphology of the cleft defect of these three cases had one common feature: the minimum width was located in the middle part of the defect in both the sagittal and vertical planes, termed the funnel type morphology. Cases with the funnel type morphology resulted in increased complexity of the bone grafting procedure in the middle part of the defect, and failed formation of a successful bone bridge in the middle part prevents tooth movement. Our aim was not to derive a formula for accurate categorization of morphology but rather to identify possible outcome associations in patients who require an optimized surgical plan because of their high likelihood of failure.

Varying SABG outcomes among different cleft morphologies suggested that an individualized surgical approach based on cleft morphology was required, rather than strict adherence to a pre-established protocol [3]. Optimizing alveolar cleft morphology via presurgical orthodontic treatment might be beneficial for SABG outcomes. Previous studies have suggested that patients with a cleft width less than 2 mm may benefit from expansion surgery before SABG. In this study, a good/moderate outcome was seen in 57.9% of cases with type IV (n = 11) morphology, whereas a poor/failure outcome was seen in 42.1% of cases with type IV morphology. At the same time, we found that the funnel part existed at the relatively palatal, occlusal area in the moderate/good result cases, while significantly narrow defect in the relatively labial, nasal area might add up the difficulty for SABG because of the worse surgical field and surgical approach, thus lead to a poor/failure result. In 19 patients with type IV morphology, the outcome included 2 failure, 6 poor, 5 moderate and 6 good results, while all failed cases had failed construction at 7 mm level. An irregular funnel type makes adequate bone grafting difficult and increases the risk of bone resorption in the funnel and adjacent areas. Orthodontic treatment before SABG in patients with type IV morphology where its funnel part existed at the relatively labial area and apical area, might improve the surgical condition for SABG afterwards. By removing the deciduous teeth adjacent to the funnel area, or moving the twisted incisor close to the cleft area towards orthodontic treatment before surgery might improve the cleft morphology and provide better surgical field and surgical approach. Types II and III were relatively rare in our study, and all such cases had a low BFR and were classified as failure/poor. Type V cases included those with extremely irregular morphology. Supernumerary, ectopic, or severely twisted teeth in the cleft site were responsible for this, and removal of these unnecessary, obstructed teeth at least 1 month before SABG (ensure the healing of extraction area) may help transform the irregular morphology to a regular type which would be beneficial for bone bridge formation.

3D radiographic imaging is superior to 2D imaging for evaluating the bony support of the teeth adjacent to the cleft [7, 32, 33], and is also reliable for assessing the volume and thickness of the bone bridge [8, 33]. In the past, satisfactory outcomes (up to 95%) had been reported in literature according to 2D evaluation methods [34], but with the consensus of using 3D evaluation methods instead of 2D in recent years, though outcomes were much poorer, it had reflected the architecture of bone defect and unsatisfactory bone formation along the labial-palatal axis [35]. 2D techniques can’t display the 3D morphology of the alveolar cleft pre- and post-surgery, and they tend to overestimate the success rate of SABG [36, 37].

Both categorical and continuous methods should be used for 3D radiographic assessments in patients undergoing SABG. The former provides an objective basis for further orthodontic treatment, and the latter provides 3D visualization of the direction of orthodontic movement. Based on the evaluation methods above, statistics showed that the outcome of SABG was not satisfactory in most cases. Only 21 patients (40.4%) demonstrated a good result, while 8 patients (15.4%) had no bone bridge formation at all assessment levels. One of the main objectives of SABG is the formation of a bone bridge, allowing tooth eruption and subsequent orthodontic tooth movement [34]. Stasiak et al. found no bone bridge in 46.43% of the measurement sites on the cleft side [27]. In this study, it was 33.2%. The results may be due to a larger sample size and the impact of different study group selection. In general, bone bridge formation exhibited better formation at the apical level (7 mm and 9 mm assessment level) compared with the occlusal level (3 mm and 5 mm assessment level). Unqualified oral hygiene maintenance after SABG or infection around the suture might add up the possibility of more bone resorption at the occlusal level. There was evidence that orthodontic tooth movement stimulates bone apposition, it was not necessary for the bone thickness to be at least the root width of the adjacent teeth [38], and bone resorption in the most inferior 1/4 level was acceptable [20]. Furthermore, in 5 patients, it was found that root length of the central incisor at the cleft side was less than 9 mm due to higher probability of malformation [39], thus bone bridge assessment at the level of 9 mm was compared with the root diameter measured 0.5 mm beneath the apex, though the scores at this level were all above 2, but they might face higher risk of root resorption in the orthodontic treatment after SABG procedure.

BFR results indicated a relatively great amount of bone resorption in most patients. A reconstruction of the cleft defect could be generated after segmentation for the surgeon and orthodontists to have a better understanding of the bony architecture [40]. A comparison of the reconstructed images of the alveolar cleft defect before and after SABG allows visualization of the resorption site [26, 36, 41]. Though BFR is the most commonly used evaluation tool for SABG procedure nowadays, a completely restored alveolar cleft defect was not the indicator of success. Some degree of bone graft resorption is compatible with a successful outcome, as long as it allows tooth eruption [42]. However, percentage ratios do not provide a spatial assessment of the bone bridge architecture [27], whether orthodontic movement would be available afterwards could not be decided according to BFR only.

Although some authors considered 9–11 years was the optimal age for SABG, however, the practice of early secondary bone grafting was not used in many departments mainly because of the concern about the possibility of subsequent maxillary growth restriction [43]. The study was not able to find any correlation between patient age and SABG outcome at the statistically significant level. It is possible that the age range of our material was too narrow (8–18 years at the procedure) to register any statistically significant results. Hence, it does not seem to be in contradiction to the studies registering age-related correlation with the SABG outcome [15, 44]. If the initial cleft size is relatively large, distraction osteogenesis may be an alternative [11].

Compared with previous reports, this study presented a new method for the classification of alveolar cleft morphology. By combining the categorical and continuous evaluation methods together, enabled a more precise examination of the outcomes of SABG. These evaluations provide further information on the morphology and need for multi-disciplinary treatment based on the morphology of the cleft defect. Despite the possibilities of orthodontic movement after SABG in some patients, bone defects still existed in the majority of cases. Slow orthodontic movement with strict periodontal control is always suggested.

## Conclusion

The new method for the classification of alveolar cleft morphology summarized in this study was comprehensive and convenient for clinical application. Both categorical and continuous methods should be used for radiographic assessments in patients undergoing SABG. The chances of a successful procedure might be better when the patient has type I or IV morphology, in which the shape is like a funnel in the relatively palatal or occlusal area towards the vertical dimension. A relatively great amount of bone resorption was observed in most patients.

## Data Availability

The data that support the findings of this study are available from Peking University School and Hospital of Stomatology, but restrictions apply to the availability of these data, which were used under license for the current study, and so are not publicly available. Data are however available from the authors upon reasonable request and with permission of Peking University School and Hospital of Stomatology.
